# Resurrection Plants—A Valuable Source of Natural Bioactive Compounds: From Word-of-Mouth to Scientifically Proven Sustainable Use

**DOI:** 10.3390/metabo14020113

**Published:** 2024-02-07

**Authors:** Dimitar Djilianov, Daniela Moyankova, Petko Mladenov, Tanya Topouzova-Hristova, Aneliya Kostadinova, Galya Staneva, Diana Zasheva, Strahil Berkov, Lyudmila Simova-Stoilova

**Affiliations:** 1 Agrobioinstitute, Agricultural Academy, 8 Dragan Tzankov Blvd., 1164 Sofia, Bulgaria; dmoyankova@abi.bg (D.M.); mladenovpetko@yahoo.com (P.M.); 2 Faculty of Biology, Sofia University ‘St. Kliment Ohridski’, 8 Dragan Tzankov Blvd., 1164 Sofia, Bulgaria; topouzova@biofac.uni-sofia.bg; 3 Institute of Biophysics and Biomedical Engineering, Bulgarian Academy of Sciences, Acad. Georgi Bonchev Street, Bl. 21, 1113 Sofia, Bulgaria; anik@bio21.bas.bg (A.K.); gstaneva@bio21.bas.bg (G.S.); 4 Institute of Biology and Immunology of Reproduction, Bulgarian Academy of Sciences, Tsarigradsko Shosse, 73, 1113 Sofia, Bulgaria; zasheva@ibir.bas.bg; 5 Institute of Biodiversity and Ecosystem Research, Bulgarian Academy of Sciences, 23 Acad. Georgi Bonchev Street, 1113 Sofia, Bulgaria; berkov_str@yahoo.com; 6 Institute of Plant Physiology and Genetics, Bulgarian Academy of Sciences, 21 Bldg. Acad. Georgi Bonchev Street, 1113 Sofia, Bulgaria; lpsimova@yahoo.co.uk

**Keywords:** resurrection plants, secondary metabolites, bioactive compounds, myconoside, *Haberlea rhodopensis*

## Abstract

Resurrection plant species are a group of higher plants whose vegetative tissues are able to withstand long periods of almost full desiccation and recover quickly upon rewatering. Apart from being a model system for studying desiccation tolerance, resurrection plant species appear to be a valuable source of metabolites, with various areas of application. A significant number of papers have been published in recent years with respect to the extraction and application of bioactive compounds from higher resurrection plant species in various test systems. Promising results have been obtained with respect to antioxidative and antiaging effects in various test systems, particularly regarding valuable anticancer effects in human cell lines. Here, we review the latest advances in the field and propose potential mechanisms of action of myconoside—a predominant secondary compound in the European members of the Gesneriaceae family. In addition, we shed light on the possibilities for the sustainable use of natural products derived from resurrection plants.

## 1. Introduction

Plants have been the main source of food, feed, and energy since the very establishment of human civilizations. At the same time, the use of plants’ parts, derivatives, or infusions for other aspects of human welfare also dates back to the roots of human history. Despite the enormous recent progress in natural science, there is still not enough knowledge about extracted plant metabolites and their mode of action when applied to various biological objects. However, positive correlations between the antioxidant properties of numerous plant extracts and infusions and their beneficial potential for human welfare, including medicine, cosmetics, food additives, etc., could be easily made [1]. It is widely accepted that plants’ survival under unfavorable environments is based on, or at least involves, tolerance to oxidative stress [2].

Studies on the mechanisms of environmental stress tolerance of plants could be a good prerequisite and a reason for parallel or consecutive testing of their metabolites as potential bioactive compounds for human benefits. In this respect, the so-called resurrection plants appear to be a very useful model.

Resurrection plants or plants with vegetative desiccation tolerance are a group of higher species able to withstand the drastic decrease in their vegetative tissues’ water content to an almost dry state, and, after long periods of dryness, to recover fast (within hours or 1–2 days) and fully when water is available again [3]. Despite the fact that angiosperm resurrection plants account for less than 0.1% of all higher plant species worldwide, they belong to several botanical families and can be found on every continent, except Antarctica, with habitats in different climate zones and at various altitudes. As could be expected, most of them live under desert or semi-desert conditions. However, there are also species that belong in humid tropical regions in Africa and South America or survive winters with freezing temperatures in Europe [3,4,5,6,7,8,9,10,11]. Their strategies to withstand desiccation are predetermined, constitutive (e.g., in-advance high-abundance of protective compounds, including metabolites), and/or inducible, leading to reprogramming at the transcriptome and metabolome levels upon stress establishment [4,12,13,14,15,16,17].

To survive and recover after extreme water deficit, resurrection plants have evolved complex strategies, including dynamic changes of primary and secondary metabolites [18]. In parallel, the metabolism of these plants attracts additional attention to them as potential sources of compounds with various applications [19,20,21]. Moreover, in comparison with typical medicinal plants, the resurrection species accumulate and/or maintain valuable bioactive compounds in the highest concentrations under desiccation. The accumulated metabolites persist for long periods, which is a good prerequisite for using long-term stored dried samples as sources for further extractions [22].

There is no sound scientific evidence for the ethnobotanical word-of-mouth data related to the potential use of resurrection plants in folk medicine in Eastern Europe or the Pyrenees against human bronchitis, diarrhea, liver diseases, pneumonia, and infectious diseases, or foot-and-mouth disease in livestock [23,24,25].

It appears that probably the only direct connection between indigenous people’s knowledge about resurrection behavior of some local plant species and well-established traditions for using them in folk medicine and religious rituals and current attempts for utilization occurs in southern Africa [26,27]. There are examples of the traditional use of *Myrothamnus flabellifolius* aqueous extracts or tea infusions against life-threatening conditions or cases of depression and mental disorders [28], as well as in the treatment of a wide range of various other diseases [26,29].

The review, published about ten years ago [30], paved the way towards the potential of resurrection plants as sources of natural products with eventual valuable applications. In the meantime, numerous intensive studies in this field have been performed. Our aim in this article is to review the recent advances in the application and mode of action of bioactive compounds isolated from resurrection species and to shed light on the possibilities for their sustainable use.

## 2. Metabolite Profiling and Application of Resurrection Plant Extracts as Bioactive Compounds

Metabolic profiling of resurrection plants is performed by applying contemporary extraction procedures and analytical methods and machinery [21]. With respect to primary metabolism, it is widely accepted that sugars are among the major players in the plant desiccation tolerance complex [18,21]. The dynamics of both sucrose and raffinose family oligosaccharides (RFOs) is well-outlined [31,32], and the specific key role in some resurrection species of relatively rare molecules—e.g., octolose, stachyose, and trehalose—is also discussed [33,34,35]. The high amounts of sugars explain the proposed inclusion of *Myrothamnus flabellifolius*, the most studied African resurrection species, as a still underestimated but potential source of nutraceuticals [26]. Due to the high amounts of carbohydrates, particularly trehalose, raffinose, and stachyose, along with sucrose, the inclusion of crude extracts in poultry diets has been suggested. Future studies should be performed with a focus on the doses of crude extracts that are added to the feed, since negative effects, such as DNA and chromosomal damage, cell damage, and mutagenic activity, may possibly be observed [36,37].

The data on secondary metabolism in resurrection plants are much more limited, at least in part due to the fact that these plants are still not regarded as sources of valuable natural compounds [21]. The few exceptions available—*M. flabellifolius*, and the members of the Gesneriaceae (*Haberlea rhodopensis*, *Ramonda* ssp., *Boea hydrometrica*) and of the Linderniaceae (*Craterostigma plantagineum*, *Lindernia brevidens*) [32,38,39,40,41,42,43,44]—outline the high abundance and diversity of phenolics, flavonoids, etc.

**Figure 1:**
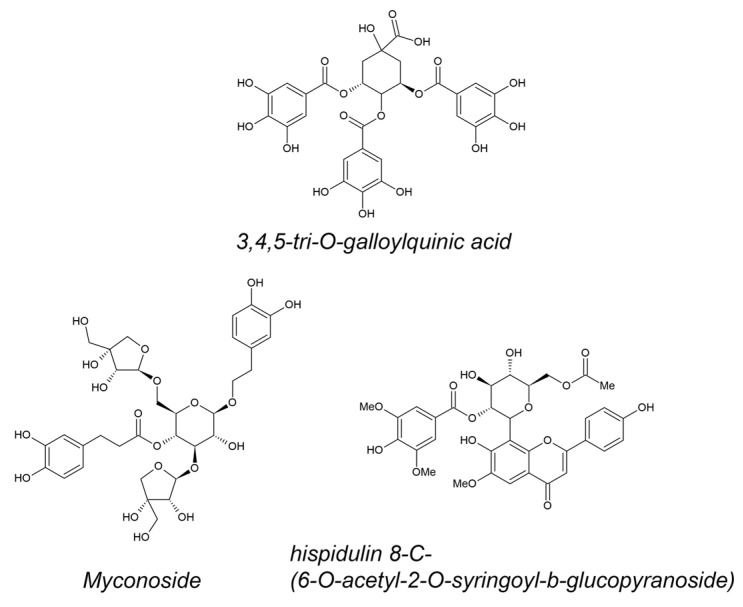
Structural formulas of 3,4,5-tri-*O*-galloylquinic acid (most abundant in *M. flabellifolius*), myconoside, and hispidulin 8-C-(6-*O*-acetyl-2-*O*-syringoyl-β-glucopyranoside) (most abundant in *H. rhodopensis*).

Unique polyphenols from resurrection plant extracts were identified in high abundance (Figure 1). The 3,4,5-tri-*O*-galloylquinic acid was identified as a predominant polyphenol in *M. flabellifolius*. It is accumulated in almost twice the concentration in dry leaves in comparison with fresh [38]. The caffeoyl phenylethanoid glycoside myconoside [β-(3,4-dihydroxyphenyl)-ethyl-3,6-di-*O*-β-d-apifuranosyl-4-*O*-α,β-dihydrocaffeoyl-*O*-β-d-glucopyranoside] was isolated as the most abundant polyphenol in all members of the Gesneriaceae found in Europe, being at the same time resurrection plant species [45,46]. Hispidulin 8-C-(6-*O*-acetyl-2-*O*-syringoyl-β-glucopyranoside) was isolated for the first time recently [47] from leaves of *H. rhodopensis* as acylated hispidoline C-glicoside, possessing some unique features like 2-O-syringoyl and 6-O-acetyl moieties.

The secondary compounds play a significant role in ROS scavenging, thus underlying the desiccation tolerance of the plants [21,30]. The strong antioxidant activity of phenolic compounds also predetermines the interest in using the extracts of resurrection plants for various applications.

Studies on potential utilization of resurrection plants have predominantly been performed with crude total extracts or with their polar/apolar fractions obtained via various solvents. Such experiments are sometimes performed in parallel or hand-in-hand with characterization of the metabolic compounds and evaluation of their antioxidant activities, and efforts have been made to establish a positive correlation with the respective data available (Table 1).

Metabolic profiling and antioxidant activity studies confirm the potential of *Xerophyta* spp. as a source of crude extracts for traditional ethnomedicine [48] and pharmacological application [49]. *M. flabellifolius* is also rich in polyphenols, especially flavonoids, and its extracts effectively suppress the growth of the leukemic cell line HL-60, but not the non-leukemic lymphocytes of the TK6 line [50]. Fractionation of the same plant species’ extracts resulted in active growth suppressing of the triple negative breast cancer cells (TNBCs) from two cell lines, BT-549 and MDA-MB-231, compared to the normal MCF10-A cell line. The main component of the efficient fraction was identified as a derivative of galloyl glucose hexahydroxydiphenic acid called strictinin (chemical name: 3-*O*-galloyl-4,6-[(*S*)-hexahydroxydiphenoyl]-b-glucopyranose). Later, strictinin was shown to suppress the activity of Receptor Tyrosine Kinase Orphan-like 1 (ROR1), which is highly active during embryonic development but is not found in growing tissues except in some tumors. In this case, the likely antitumor action is associated with reduced phosphorylation of the AKT kinase and increased apoptosis [66,74]. In another study, the main polyphenol isolated from *M. flabellifolius*, 3,4,5 tri-*O*-galloylquinic acid, was found to inhibit HIV-1 and M-MLV reverse transcriptases and could be used as a potent antiviral drug that blocks viral replication [71].

**Figure 2:**
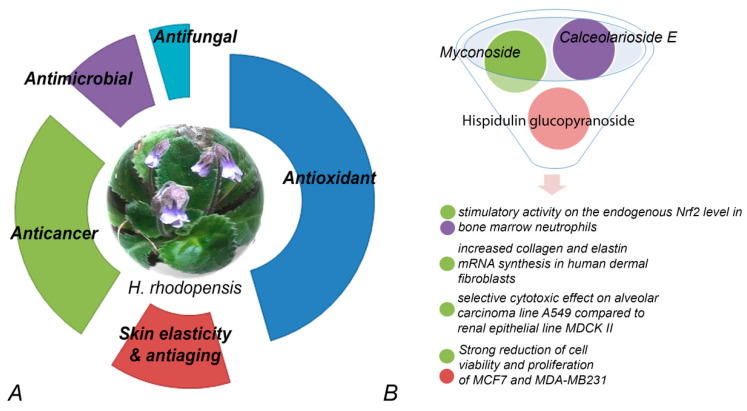
Bioactivity of extracts (**A**) and compounds (**B**) purified from *H. rhodopensis*. (**A**) Percentage of relative contribution of published studies on the various biological activities of extracts. (**B**) Myconoside, hispidulin 8-C, and calceolarioside E have been enriched or purified from different fractions. Fractions containing pure myconoside or myconoside in combination with the other two compounds have significant effects on various cell lines.

So far, our review of the available literature shows that the potential applications of bioactive compounds isolated from resurrection plants have been predominantly studied in the Balkan endemic plant *H. rhodopensis* (Table 1). Data for the biological activity of total extracts or purified compounds are summarized in Figure 2.

There was very weak or even no biological activity found in some experimental systems, e.g., no direct virus inactivating effect was found in HSV (*Herpes simplex* virus) [59], and there was a lack of anticancer effect in some cell lines [65] or very poor antimicrobial activity [63]. On the other hand, promising results were reported when *H. rhodopensis* extracts were tested as protectors to minimize the harmful effects of radiotherapy [75]. The use of ionizing radiation is one of the widely used approaches in treating various cancers. However, quite often, negative side effects appear during such treatments as a result of the oxidative stress that irradiation has on genomic DNA, lipids, proteins, enzymes, and membranes of living organisms. In this respect, the search for nontoxic and efficient radioprotectors, particularly of plant origin, is very intensive, since a high positive correlation was reported between phenolic compound contents and their antioxidant capacity. An interesting and still not sufficiently investigated issue is the potential use of extracts from resurrection plants’ tissues to manipulate the reaction of higher living organisms to ionizing radiation [75]. Several investigations have been performed in the last 15 years where New Zealand rabbit lines were used as a platform to study the radioprotective properties of *H. rhodopensis* leaf extracts. Pre-treatment of lymphocyte cultures with such extracts reduced the numbers of aberrant cells and chromosome aberrations in a dose-dependent manner [52,53,54,55] and resulted in a reduction in induced cellular DNA damage [56]. The pre-treatment significantly increased the activity of some antioxidant enzymes and had an anti-lipid peroxidative effect by reducing MDA levels in the blood. Furthermore, a significant reduction in MN events in peripheral lymphocytes was observed. Recently [57], the preincubation of cells with *H. rhodopensis* extracts was shown to modulate HeLa cancer cells’ early response to gamma IR (γ-IR) and oxidative stress. The response modulation appeared almost immediately after exposure in a dose-dependent manner, thus reducing the severity of genotoxic and oxidative stress. A strong antioxidant effect of methanol extracts of *H. rhodopensis* was shown in both non-neoplastic and prostate cancer cells, where the extracts reduced H_2_O_2_-generated oxidative stress [61]. The pretreatment of non-malignant cell line HEK 293 was apoptosis-protective and cell death-reducing when H_2_O_2_-induced oxidative stress was applied. NFκB was activated in p53^+/+^ cells and suppressed in p53^−/−^ cells. Leaf water and ethanol extracts were applied to a range of other human cancer cell lines [62]. Water extracts were reported not to be antiproliferative, while ethanol extracts were particularly effective to hepatocellular carcinoma (HepG2) and non-small cell lung adenocarcinoma (A549) cell lines and were found to exert significant antimigratory concentration-dependent effects in both cell lines.

Total methanol extracts and polar and apolar fractions were tested in a completely different system—important plant pathogens to search for potential sustainable and eco-friendly plant protection approaches [60,64]. No fungitoxic effect on *Alternaria alternata* and *Fusarium oxysporum* was found. Strong inhibition of *Botrytis cinerea* was achieved, in particular by apolar fractions. The same fractions stimulated the growth of *Phytophthora citricola* and can be potentially used as an effector. Other *Phythophtora* spp. isolates were stimulated significantly to grow under in vitro conditions, which could be a good prerequisite for the development of culture media for further tests on these obligate pathogens.

The significant amount of myconoside in plant extracts from European Gesneriads along with the strong scavenging activity gave ground for potential application in various human welfare areas. There is a significant interest in the application of myconoside as a single compound or in well-characterized combination with other secondary metabolites in various medicinal test systems.

An extract rich in myconoside isolated from *H. rhodopensis* was reported to increase mRNA synthesis of collagen and elastin genes in human dermal fibroblasts stressed with H_2_O_2_ [70]. The extracts were proven to protect against UV-induced dermis oxidation and even increased skin elasticity of human volunteers. It was suggested that *H. rhodopensis* extracts can be used for anti-aging treatments, protecting the skin from oxidation, increasing skin elasticity, and enhancing skin radiance. The anti-aging potential of such extracts was further confirmed. Influence on cell periphery, permeabilization of the membrane, and disruption of HaCaT keratinocyte tight junctions were observed—more pronounced in actively dividing cells [58]. In a very different experimental system, the strong and specific proliferative, anti-aging, and protective effect was found in the model yeast *Saccharomyces cerevisiae* cell line to revitalize and ameliorate cellular growth as well as to balance intracellular metabolic states [51]. Myconoside, isolated from *H. rhodopensis* extracts, was shown to have strong antioxidative potential along with a significant hepatoprotective effect on isolated rat hepatocytes [68]. *H. rhodopensis* extract fractions, containing myconoside or enriched with Calceolarioside E, were found to be very effective in promoting the expression of nuclear factor erythroid 2 p45-related factor 2 (Nrf2), a transcriptional regulator of the cellular redox balance and a suppressor of the pathological manifestation of various diseases, in bone marrow neutrophils [69]. 

## 3. Potential Mechanisms of Action of the Phenolic Glycoside Myconoside

Many studies in in vitro model systems have shown pronounced anticancer activity of *H. rhodopensis* extracts, but the molecular mechanisms of this action are largely unclear. Recent studies gave ground to the proposal of potential mechanisms of action for myconoside—the predominant polyphenol in *H. rhodopensis* and *Ramonda* spp.

Investigations on model lipid membranes confirm that polyphenols can penetrate the lipid bilayer to different depths, depending on their structure and physicochemical properties [76,77]. The hydrophobicity of polyphenols decreases with the increasing number of hydroxyl groups and the presence of glycosidic substituent [78]. Myconoside possesses 11 hydroxyl groups and a glycosidic residue, suggesting that it exhibits an amphiphilic affinity and will have different effects on cellular membranes with various overall fluidity. The action of myconoside was investigated in two cell lines with pronounced differences in membrane fluidity—the alveolar carcinoma line A549, which is characterized by more fluid membranes, and the non-cancerous renal epithelial line MDCK II, which is characterized by a high degree of order of membrane lipids, the presence of stable intercellular contacts, and the ability to polarize in the epithelial monolayer. The results of the cell studies were compared with experiments on biomimetic membranes [72,73]. A selective cytotoxic effect was observed for A549 at high concentrations of myconoside, in comparison to the absence of such in the MDCKII cell line. This is probably due to a dual effect on cancer cells depending on the applied concentration: a strong inhibitory effect by reducing the lipid order of the plasma membrane and damage to the actin cytoskeleton by affecting its connection with the plasmalemma. In biomimetic membranes, myconoside acts as a molecule stimulating or destroying raft-type domains, depending on its concentration. Such a dependence is logical given the effect that myconoside exerts on the arrangement of lipids in the membrane depending on its concentration. At low concentrations, myconoside probably acts as a molecular filler, occupying the vacant interlipid spaces between the glycerol residue and the polar head, reducing the number of water molecules in this region. This mode of interaction of myconoside with lipids leads to a higher order of the membranes, which stimulates the formation of more ordered raft domains. At high concentrations, when the interlipid spaces around the glycerol are filled, myconoside accumulates increasingly, primarily between and above the polar heads, forming myconoside clusters. Thus, it reduces the interaction between lipids, playing the role of a molecular separator, reducing the order of lipids in the membrane, and preventing the formation of ordered raft domains [72,73]. A similar mechanism was observed for different types of flavonoids, but not for a given type of flavonoid and as a function of its concentration [79].

In addition to lipids, flavonoids and glycoside flavonoids can also interact with certain membrane proteins, especially those that have significant hydrophobic regions. Recently, a possible interaction of myconoside with the GLUT1 glucose membrane transporter, which is overexpressed in several cancer cell lines, and the estrogen receptor was proposed [67]. This transmembrane protein belongs to a family of facilitative transporters that transport hexose molecules down the concentration gradient in a tissue- and substrate-specific manner [80].

Applying molecular docking analysis, it was proposed that myconoside is inserted into the central part of the transporter, probably with the simultaneous participation of hydrophobic and hydrophilic interactions [67]. The binding of myconoside probably blocks or at least reduces the uptake of glucose into cancer cells, thereby significantly inhibiting their growth in vitro. The amphiphilic nature of myconoside makes a similar interaction with other membrane receptors specific for steroid hormones possible, thus influencing the signaling pathways associated with them. Simultaneously, affecting the organization of membrane lipids and the activity of the associated membrane proteins can lead to drastic changes in membrane organization and functions.

Lipid rafts are dynamic nanoscale molecular platforms enriched in cholesterol and sphingolipids in cell membranes’ inner and outer bilayers. They form functional platforms for the regulation of cellular processes. Secondary plant metabolites have been suggested to be capable of interacting with lipid rafts in two ways. First, they can disrupt the integrity of lipid rafts by altering their structure and organization, which leads to rearrangement (aggregation) of the raft domains. It was shown that myconoside isolated from *H. rhodopensis* can increase or decrease the fraction of raft domains in a concentration-dependent manner in biomimetic systems [72,73]. A second molecular mechanism by which plant metabolites can modulate downstream signaling pathways mediated by lipid rafts is by binding to receptor proteins localized to lipid rafts such as the 67 kDa laminin receptor (67LR), the epidermal growth factor receptor (EGFR), disruption of cytoskeleton integrity, and others [79]. In cancer cells, increased amounts of laminin receptors are found in the raft domains of the plasma membrane, which is associated with the spread of tumor metastases. Secondary plant metabolites may reduce the likelihood of carcinogenesis by blocking the interaction of the epithelial growth factor (EGF) with the corresponding receptor (EGFR), which localizes precisely in the raft domains [81].

Interestingly, secondary plant metabolites can interact directly with actin. Despite the similar values of binding constants of structurally related flavonoid molecules to actin, it has been demonstrated, for example, that flavonols inhibit actin functions while the flavan epigallocatechin stimulates its activity [79]. Other cellular raft domains, such as caveolae, are involved in caveolar endocytosis processes and are considered a special group of raft domains in the plasma membrane of various cell types. Caveolae are also rich in cholesterol and sphingomyelin and contain caveolin proteins responsible for invaginations of the plasma membrane during endocytosis [82]. It is known that some plant metabolites, such as flavonoids, can influence cell signaling and reduce inflammatory processes in endothelial cells by reducing the expression of caveolin-1 and cyclooxygenase COX-2 and inhibiting ERK ½ and Akt kinases of the MAPK signaling pathway [83]. A similar ability to reduce caveolin-1 expression and activate signaling through PI3K and Akt kinases, responsible for regulating apoptosis and carcinogenesis, was also found when cells were treated with the flavonoid daidzein [84].

Recently, it was clearly shown that treatment of two cell lines (MDCKII and cancer A549 cells) with myconoside leads to substantial changes in the F-actin and Zonula occludens (ZO-1) network associated with the accumulation of granular aggregates in the plasma membrane and in intracellular structures. However, the linear structure of F-actin and ZO-1 of MDCKII cells is much more conserved than that of A549. Higher myconoside concentration induced lower cell density, more round-shaped cells, more diffuse F-actin and ZO-1 network, and reduced cell–cell contacts in the cancer cell line [72,73].

## 4. Conclusions

At present, the data obtained after application of extracts or purified compounds derived from resurrection plants’ tissues for various purposes are still relatively limited and most of them are related to *H. rhodopensis*. However, the knowledge obtained could serve as a good background for further studies and developing strategies. Despite the low biological activity in some studies, the application of total or relatively less fractionated extracts should continue because such experiments are relatively simple and of low cost. When promising results are achieved, further efforts to isolate almost pure fractions or even compounds should be made. This means developing projects that will convince potential investors to fund the application of more sophisticated equipment and experimental design.

At this point, the issue of sustainability and biodiversity preservation should be taken into account. The standard practices for obtaining valuable compounds from medicinal plants are based predominantly on extractions from fresh samples when they are the richest of valuable compounds. With the recent climate changes, causing drought stress to become more and more frequent, the yield, regularity, and quality of raw material supply drops significantly [1]. In this respect, the increased attention to plant cell cultures as future potential biofactories of environmentally independent production of bioactive compounds highlights the achievements, problems, and challenges that are still to be solved [85]. One of the advantages of resurrection plants as sources of valuable bioactive compounds is related to the fact that these compounds are accumulated and/or maintained in high concentrations under desiccation, playing an important role in the protection of the plants. These high concentrations persist for long periods, which is a good prerequisite to use long-term stored dried samples as sources for further extractions.

In addition, some of the resurrection plants, e.g., *M. flabellifolius* and *Xerophyta* spp., are widespread and easy to access in their habitats. This also makes them a relatively cheap and sustainable source of valuable bioactive compounds. Others, like, e.g., *H. rhodopensis* and *Ramonda* spp., are endemic and belong to zones with restricted access for natural conservation. At the same time, the increasing open access to scientific data could sometimes result in inappropriate activities of some members of the society, leading to damage or even destruction of the restricted plant populations. In this respect, examples of a sustainable scientific approach, like the establishment of protocols and systems for in vitro culture, e.g., for the European Gesneriads [22,69,86,87,88], are very promising and need to be encouraged.

Resurrection plant species possess stress tolerance abilities that are almost of no match among other higher organisms. There is a broad horizon for the potential application of useful natural products, isolated from them in various areas of human welfare.

## Figures and Tables

**Table 1:** Biological activities of plant extracts and compounds from resurrection species.

Extract or Compound	Resurrection Species	Biological Effect	Ref
Crude ethanol extracts	*Xerophyta* spp.	For traditional ethnomedicine; antibacterial activity—*S. typhi*, *B. subtilis*, *S. aureus*, *E. coli*	[48]
Crude ethanol extracts	*Xerophyta* spp.	Pharmacological application for antioxidant activity	[49]
Crude ethanol, methanol and water extracts	*Myrothamnus flabellifolius*	Source of nutraceuticals	[26]
Crude methanol and petroleum ether extracts	*Myrothamnus flabellifolius*	Methanol extract suppresses human leukemic HL-60, but not non-leukemic TK6 line	[50]
Crude methanol extracts	*Haberlea rhodopensis*	Proliferative, anti-aging, and protective effect on model yeast *S. cerevisiae* cell line.	[51]
Crude ethanol extract	*Haberlea rhodopensis*	Radioprotective effects	[52,53,54,55,56,57]
Crude methanol extracts	*Haberlea rhodopensis*	Influence on cell periphery, permeabilization of the membrane, and disruption of HaCaT keratinocyte tight junctions.	[58]
Crude ethanol, methanol, water extracts, polar/apolar fractions of methanol extracts	*Haberlea rhodopensis*	Crude methanol extract was the most active in MTT assay modified for HSV. No direct virus inactivating effect.	[59]
Crude methanol extracts	*Haberlea rhodopensis*	*Phythophtora* spp. isolates were stimulated to grow under in vitro conditions.	[60]
Crude methanol extracts	*Haberlea rhodopensis*	Antioxidative effect in cancer vs. normal cell lines, and differentially modulate distinct cell lines in genotoxic and inflammatory stress.	[61]
Crude ethanol and water extracts	*Haberlea rhodopensis*	The human cancer cell lines A549, HepG2, HT29, and Caco-2 and PC3 and DU145 were treated. Water extracts—no effect. Ethanol extracts—effective to HepG2 and A459 cell lines.	[62]
Crude ethanol extracts	*Haberlea rhodopensis*	Lack of effect on *E. coli*, *S. enterica* subsp. *enterica*, *P. aeruginosa*, *S. aureus*, *B. subtilis*, *S. cerevisiae*, *A. niger*, *Rhizopus* sp., *K. pneumonia*, *L. monocytogenes*	[63]
Polar/apolar fractions of methanol extracts	*Haberlea rhodopensis*	The growth of *Botrytis cinerea* was strongly inhibited, in particular by apolar fractions. Same fraction had stimulating effect on Phytophthora citricola. No effect was found against *Alternaria alternata* and *Fusarium oxysporum*.	[64]
Polar and apolar fractions of methanol extracts	*Haberlea rhodopensis*	Polar fractions possessed strong free radical scavenging activity. No effect on HL-60, HL-60/Dox, SKW-3 (KE-37), and MDA-MB-231	[65]
Fractions of methanol extract, novel compound	*Myrothamnus flabellifolia*	Anti-triple negative breast cancer effect	[66]
Fractions rich of myconoside and hispidulin from methanol extracts	*Haberlea rhodopensis*	Significant influence on the proliferation rate of the hormone receptor expressing MCF7 and the triple negative MDA-MB231 breast cancer cell lines. No significant effects on the benign MCF10A cell line.	[67]
Myconoside and hispidulin	*Haberlea rhodopensis*	Cytoprotective, radical scavenging potential, and lipid peroxidation inhibition in rat hepatocytes.	[68]
Myconoside and Calceolarioside E	*Haberlea rhodopensis*	Increased Nrf2 expression in bone marrow neutrophils.	[69]
Myconoside-enriched fraction	*Haberlea rhodopensis*	Increases skin elasticity. Protection of human dermal fibroblasts against H_2_O_2_ damage	[70]
3,4,5 tri-O-galloylquinic acid	*Myrothamnus flabellifolius*	Inhibition of HIV-1 and M-MLV reverse transcriptases	[71]
Myconoside	*Haberlea rhodopensis*	At low concentrations—increased MDCKII cell viability by enhancing membrane lipid order and adherent junctions. Higher doses—the opposite effect.	[72]
Myconoside	*Haberlea rhodopensis*	Low concentration has no influence on human lung adenocarcinoma A549 cell viability but increases plasma membrane lipid order of the treated cells. Higher concentration inhibits cell viability.	[73]

## Data Availability

Not applicable.
